# Demand-side or supply-side stabilisation policies in a small euro area economy: a case study for Slovenia

**DOI:** 10.1007/s10663-021-09503-y

**Published:** 2021-03-22

**Authors:** Reinhard Neck, Klaus Weyerstrass, Dmitri Blueschke, Miroslav Verbič

**Affiliations:** 1 Department of Economics, Alpen-Adria-Universität Klagenfurt, Klagenfurt, Austria; 2 Institute for Advanced Studies, Macroeconomics and Public Finance Group, Vienna, Austria; 3 Faculty of Economics, University of Ljubljana, & Institute for Economic Research, Ljubljana, Slovenia

**Keywords:** Macroeconomics, Stabilisation policy, Fiscal policy, Tax policy, Public expenditure, Demand management, Supply-side policies, Slovenia, Public debt, E62, E17, E37

## Abstract

In this paper we analyse the effectiveness of demand- and supply-side fiscal policies in the small open economy of Slovenia. Simulating the SLOPOL10 model, an econometric model of the Slovenian economy, we analyse the effectiveness of various categories of public spending and taxes during the period 2020 to 2030, assuming that no crisis occurs. Our simulations show that those public spending measures that entail both demand- and supply-side effects are more effective at stimulating real GDP and increasing employment than pure demand-side measures. This is due to the fact that supply-side measures also increase potential and not only actual GDP. Measures which foster research and development and those which improve the education level of the labour force are particularly effective in this respect. Employment can also be stimulated effectively by cutting the income tax rate and the social security contribution rate, i.e. by reducing the tax wedge on labour income, which positively affects Slovenia’s international competitiveness. Successful stabilisation policies should thus contain a supply-side component in addition to a demand-side component. We also provide a first simulation of potential effects of the Covid-19 crisis on the Slovenian economy, which is modelled as a combined demand and supply shock.

## Motivation

Until recently, the financial and economic crisis of 2007 to 2009, meanwhile known as the Great Recession, was the most severe economic crisis since the Great Depression of the 1930s. In Slovenia, real GDP declined by as much as 7.8 percent in 2009. During the “Great Moderation” from the mid-1980s onwards (Lucas 2003), most economists and international organisations like the IMF or the OECD had more or less agreed that the optimal fiscal policy stance would be to refrain from discretionary policies and to only let automatic stabilisers operate. During the Great Recession, monetary policy reacted quickly, by introducing expansionary measures, and fiscal policies followed suit, letting automatic stabilisers work and also introducing discretionary tax reductions or increases in public expenditure. In the Euro Area, the only macroeconomic stabilisation policy instrument available to Euro Area members was fiscal policy. It is, therefore, of interest to investigate the effectiveness of fiscal policy in stabilising an economy like Slovenia’s. Such an analysis is even more interesting since in academia there is no consensus about the effectiveness of expansionary fiscal policy measures. While some authors (e.g. Taylor 2009) argue against discretionary fiscal policy, others are more in favour of tax reductions or expenditure increases and expect potentially large multiplier effects (e.g. Romer and Romer 2010). In view of the architecture of the Euro Area and the fact that most of its members are small open economies, it is important and of general interest to clarify the appropriate role of fiscal policy for small open economies in a monetary union which are constrained by the problem of high public debt.

In this paper, we aim at contributing to this debate by empirically estimating fiscal policy effects for the Euro Area economy of Slovenia. We are particularly interested in the question as to whether demand-side (Keynesian) fiscal policies aiming primarily at supporting aggregate demand can contribute to stabilising this economy or whether policies primarily addressing the supply side are more successful. The debate between Keynesians and supply siders has been a hot topic since the second half of the 1970s, and (like many macroeconomic policy debates) it has not yet been completely settled. The prevailing opinion (albeit not a consensus) considers demand-side policies to be appropriate when combating an adverse demand-side shock but not necessarily when faced with a supply-side shock. In the Great Recession, even the IMF, which had been very cautious towards demand-side policies before, argued in favour of supporting demand-side policies. Nevertheless, for the medium and longer term in particular, the European Commission proposed (and in some cases prescribed to member states) structural reforms to enhance potential growth. This implies that fiscal policy should also include supply-side measures. Such prescriptions are still issued regularly by the European Commission and other institutions today. In contrast, many politicians and interest group representatives heavily criticised what they called the “austerity regime” of the Commission and advocated an expansionary fiscal policy stance in spite of already high public debt.

Within academia, advocates of different macroeconomic theories still diverge in their judgment of the effectiveness of fiscal policies during a recession. In particular, the role of fiscal policy and the specific problems of countries within the Euro Area are the subject of ongoing controversies (see, for instance, Coenen et al. 2008; Cogan et al. 2010). It is well known that fiscal policy effects are smaller ceteris paribus in small and open economies than in larger and less open economies. Slovenia is an interesting case because it is the only small open transition economy which was already in the Euro Area before the Great Recession. Furthermore, an already high level of public debt is likely to undermine any positive effects of fiscal stimuli. Hence, a clear commitment to fiscal consolidation after overcoming a crisis is required (see, e.g., Spilimbergo et al. 2009; IMF 2008). Fiscal multipliers may not only depend on the openness of an economy, but also on its position in the business cycle. Auerbach and Gorodnichenko (2013) conclude that in particular spending multipliers tend to be larger in recessions than in expansions. Furthermore, strict fiscal consolidation measures in a recession might contribute to a deepening of the recession (Blanchard and Leigh 2013).

In this paper, we analyse the effects of different fiscal policy measures in Slovenia. We use the SLOPOL model, an econometric model of the Slovenian economy which we constructed, to simulate the effects of various tax and spending policies on important macroeconomic variables as well as on the public debt level. These simulations extend earlier simulations reported in Blueschke et al. (2019), Weyerstrass et al. (2020), and Neck et al. (2018) by focusing on some supply-side components of fiscal policies in addition to their demand-side effects.

Here we examine the question as to whether Slovenia would benefit more from a demand- or supply-side orientation of its fiscal policy with the help of an econometric model. The plan of the paper is as follows: Sect. 2 describes the macroeconometric model SLOPOL10 which is used for the empirical analysis. Section 3 presents a forecast of the Slovenian macroeconomy for the years 2020 to 2030 obtained with the model and assuming no further exogenous shock in this period, which serves as the baseline solution for the policy simulation. In Sect. 4, we analyse fiscal policies in several model simulations. It turns out that expenditure-side budgetary measures with a strong supply-side content (especially research and development related spending and the enhancement of human capital) are most successful and effective at stabilising the Slovenian economy, while tax policies exert much smaller and transitory effects, except on unemployment. Section 5 presents a first tentative simulation of the Covid-19 (Corona virus) shock which is affecting Slovenia (and virtually all countries in the world) at present. Section 6 concludes.

## The macroeconometric model SLOPOL10

For this study we used an updated version of SLOPOL, a medium-sized macroeconometric model of the small open economy of Slovenia. In the most recent version, SLOPOL10 consists of 75 equations, 23 of which are behavioural equations and 52 identities. In addition to the 75 endogenous variables, the model contains 41 exogenous variables. A more detailed discussion of the model and its properties is given in Weyerstrass et al. (2018).

The behavioural equations were estimated by ordinary least squares (OLS), except for the labour demand and supply equations, which were estimated as censored Tobit models. Stationarity tests indicate that the vast majority of the variables are either stationary or cointegrated. Hence it was decided to specify the equations in error correction form, with only a few exceptions. The results of the unit root and cointegration tests can be found in Weyerstrass et al. (2018).

The behavioural equations were estimated using quarterly data for the period 1995q1 to 2015q4. Data for Slovenia and for Euro Area aggregates as well as the oil price were taken from the Eurostat database, and world trade data came from the CPB Netherlands Bureau for Economic Policy Analyses. For this paper, the database has been updated to 2019q4 but the equations were not re-estimated.

The model contains behavioural equations and identities for the goods market, the labour market, the real effective exchange rate, the money market (albeit only rudimentary) and the government sector. The model combines Keynesian and neoclassical elements. In the short run, the model is demand driven, while in the long run the growth path is determined from the supply side via potential output. In the following, the model equations are described.

The supply side incorporates neoclassical features. In accordance with the approach applied by the European Commission for all EU member states (Havik et al. 2014), potential output (full-employment or potential GDP) is determined by a Cobb–Douglas production function with constant returns to scale. Potential output is determined by trend employment (the labour force minus structural unemployment), capital stock and the trend of total factor productivity. In line with the literature on production functions as well as international practice in macroeconometric modelling, the elasticities of labour and capital were set at 0.65 and 0.35 respectively. These elasticities correspond approximately to the shares of wages and profits, respectively, in national income. The NAIRU, which approximates structural unemployment, is taken from the estimate provided by the European Commission. It was extracted in June 2019 from the website of the European Commission on which the results of its economic forecasts are published.

Several steps are required to determine technical progress. Firstly, ex-post total factor productivity (TFP) is calculated as the Solow residual, i.e. that part of the change in GDP that is not caused by changes in the production factors of labour and capital, weighted with their respective production elasticities. In a second step, the trend of technical progress is then determined by applying the Hodrick-Prescott (HP) filter. For simulations and forecasts, the trend of the TFP is explained in a behavioural equation. In accordance with the literature on endogenous growth, technical progress is influenced by the proportion of people with tertiary education in the labour force. In addition, trend TFP is influenced by the real investment ratio as well as lagged real government spending on research and development (R&D).

On the demand side, the consumption of private households is explained by a combination of a Keynesian consumption function and a function in accordance with the permanent income hypothesis and the life cycle hypothesis. Thus, private consumption depends on current disposable income and on the long-term real interest rate, the latter entering the consumption equation with a negative sign. Real gross fixed capital formation is influenced by the change in total domestic demand (in accordance with the accelerator hypothesis) and by the user cost of capital, defined as the real interest rate plus the depreciation rate of capital stock. Changes in inventories are treated as exogenous in the SLOPOL model, as in many macroeconomic models in use around the world.

Real exports of goods and services are a function of the real exchange rate and foreign demand for Slovenian goods and services. Foreign demand is approximated by the volume of world trade. Real imports of goods and services depend on domestic final demand and the real exchange rate.

Labour demand and labour supply are divided into the main age group (15 to 64 years) and older people (65 years and above). The labour demand of companies, i.e. actual employment, is modelled via the employment rates of the two age groups, i.e. employment as a share of the relevant age group in the total population. Both equations were estimated as Tobit models, the employment rates being restricted to lie between 0 and 0.9 (15 to 64 years) and between 0 and 0.5 (65 years and older) respectively. Both employment rates are influenced positively by real GDP and negatively by the real net wage and additionally by the wedge between the gross and the net wage. A higher tax wedge is borne partly by employers and partly by employees, depending on the bargaining position of the two sides of the labour market. Labour supply is modelled via the share of the labour force of the two age groups in the total population. These equations, too, were estimated as Tobit models with the restriction of being positive but below 0.95 and 0.9 respectively. Labour supply depends positively on the real net wage and negatively on the wedge between the gross and the net wage.

In the wage-price system, gross wages, the consumer price index (CPI) and various deflators are determined. The gross wage rate depends on the price level, labour productivity and the unemployment rate. This equation is based on a bargaining model of the labour market, where the relative bargaining power of the employees (or the trade unions) is negatively affected by unemployment. The CPI is linked to the private consumption deflator, the latter depending on the import deflator, unit labour costs and the capacity utilisation rate. The inclusion of the capacity utilisation rate in the price equation represents a channel for closing an output gap by increasing (reducing) prices in the case of over-utilisation (under-utilisation) of capacities. The public consumption deflator is linked to public consumption, which includes purchases of goods and services and the wage costs of public employees. In analogy to consumer prices, both the investment and export deflators are influenced by domestic and imported cost elements. The former are approximated by the unit labour costs in the investment deflator equation and the gross wage rate in the export deflator equation respectively while the latter are captured by the import deflator. Finally, the import deflator is influenced by the oil price in euros as a proxy for international raw material prices.

On the rudimentarily modelled money market, the short-term interest rate is linked to its Euro Area counterpart so as to capture Slovenia’s Euro Area membership and the resulting gradual adjustment of interest rates in Slovenia towards the Euro Area average. In the same vein, the long-term Euro Area interest rate is included in the equation determining the long-term interest rate in Slovenia. In addition, the long-term interest rate is linked to the short-term rate, representing the term structure of interest rates. Furthermore, the long-term interest rate is influenced by the debt-to-GDP ratio, representing a risk premium that rises with the debt ratio. The foreign exchange market is modelled by the real effective exchange rate against a group of 41 countries. When determining the effective exchange rate for Slovenia, the fact that the country has only been a Euro Area member state since 2007 has to be taken into account. As the time series on which the estimations of the behavioural equations are based include the period before Slovenia’s Euro Area accession in 2007, the bilateral exchange rate between the Slovenian tolar and the euro is included as one of the explanatory variables in the real effective exchange rate equation. In addition, the exchange rate between the euro and the US dollar is considered as well as inflation in Slovenia.

In the government sector block of the model, the most important expenditure and revenue items of the Slovenian budget are determined. Social security contributions by employees are calculated by multiplying the average social security contribution rate by the gross wage rate and the number of employees. In the same vein, income tax payments by employees are determined by multiplying the average income tax rate by the gross wage rate and the number of employees. In a behavioural equation, social security payments by companies are linked to social security contributions by employees. Profit tax payments by companies are explained by GDP as an indicator for the economic situation. Value added tax revenues depend on the value added tax rate and private consumption. Other direct and indirect taxes are determined via their relation to nominal GDP. Interest payments on public debt depend on the lagged debt level and the long-term interest rate. Public consumption, transfer payments to private households and the remaining public expenditures and revenues are exogenous. The public debt level is extrapolated using the budget balance equation. The model is rounded off by a number of identities and definition equations.

Although the SLOPOL model is used for forecasting and policy simulations, it should be noted that the model—like every structural econometric model—may be subject to the famous Lucas critique. Lucas (1976) argued that the relations between macroeconomic aggregates in an econometric model should differ according to the macroeconomic policy regime in place. In this case, the effects of a new policy regime cannot be predicted using an empirical model based on data from previous periods when that policy regime was not in place. As Sargent (1981) argues, the Lucas critique is partly based on the idea that the parameters of an observed decision rule should not be viewed as structural. Instead, the equations should only contain “deep parameters” such as preferences and technologies. These parameters would be invariant to changes in policy regimes. Providing for such “deep parameters” requires Computable General Equilibrium (CGE) or Dynamic Stochastic General Equilibrium (DSGE) models. Performing simulations with similar policy instruments as used here with such models might be useful to obtain information about the sensitivity of our results with respect to the underlying theories.

An approach taking the Lucas critique into account in structural models like SLOPOL emerged in the so-called London School of Economics tradition initiated by Sargan (1964). According to this approach, economic theory guides the determination of the underlying long-run specification while the dynamic adjustment process is derived from an analysis of the time series properties of the data series. Error correction models involving cointegrated variables combine the long-run equilibrium and the short-run adjustment mechanism.

A further argument for the robustness of our estimations can be seen in the fact that we did several updates of the model when preparing this paper and found that it does well when making out-of-estimation-period forecasts without changing the estimated equations. In addition, the calculations of the multipliers presented in Sect. 4 below give virtually the same results as do calculations for the years immediately after the estimation period. Hence, the model can be regarded as a reliable tool for forecasting the development of the Slovenian economy in the short and medium run.

## A medium-run “business as usual” projection of the Slovenian economy

We use the SLOPOL10 model to simulate different discretionary fiscal policies over the period 2020 to 2030. As no sign of a deep recession (as caused by Covid-19) could be seen at the beginning of 2020, we use assumptions about the future economic environment in “normal times”, i.e. a “business as usual” scenario. Possible effects of Covid-19 will be investigated in Sect. 5. Here the impacts of the policy measures on important macroeconomic indicators like GDP growth, inflation, unemployment, net exports and public finances are determined by means of deviations of the policy scenarios from this baseline with policies “as usual”. We mostly used the autumn 2019 forecast of the European Commission for Slovenia (European Commission 2019) to construct this baseline scenario.

The baseline simulation requires assumptions on the future developments of the international and domestic non-policy exogenous variables as well as on the trajectories of the policy instruments. The international variables comprise world trade, the oil price and Euro Area interest rates. Slovenian variables that are largely beyond the control of the policy makers are mainly the population in the different age groups while Slovenian policy instruments comprise tax rates and various government spending items.

For the interest rates, we assumed that the European Central Bank would start to raise its policy rates in the second half of 2020 at the earliest. Hence the three-month Euribor was assumed not to become positive before 2021. Afterwards it would continue to rise gradually to reach slightly less than 2 percent in 2030. Based on the term structure of interest rates, this implies that the Slovenian long-term interest would only increase at a very modest pace as well. The exchange rate between the euro and the US dollar was held constant at 1.14 dollars per euro. During the first few months of 2019, world trade was very sluggish. Hence, we assumed that it would only grow at 3.1 percent in 2020, 3.5% in 2021 and 4% p.a. from 2022 onwards. For the oil price (Brent), we assumed a moderate growth rate of 1 percent p.a. from 2020 on.

According to projections by the Slovenian Statistical Office, Slovenia’s working-age population will decline by about 6 percent by 2030. Conversely, as is the case all over Europe, the population aged 65 and over will continue to rise.

Turning to the fiscal policy instruments, it was assumed that the tax and social security contribution rates would not be changed from their 2019 values. Government consumption, public investment in equipment and machinery, public spending on research and development, transfer payments to private households and residual government expenditures and revenues were all assumed to increase by 4 percent p.a. from 2019 until the end of the simulation period.

These settings of the exogenous variables lead to the following baseline simulation results until 2030. For 2020 our model predicts real GDP growth of 2.7 percent and afterwards stabilising at about 2 percent per year. Due to the projected population development and relatively low GDP growth, employment is forecast to decline somewhat until the end of the simulation period. The consumer price index inflation rate is forecast to decrease and to stabilise at 1 percent p.a. Due to favourable economic developments, the Slovenian budget experienced a surplus in 2019. According to our model prediction, the surplus will prevail until 2029, with a small deficit in 2030. As a consequence of this development of the budget balance, the debt-to-GDP ratio will decline from 66 percent in 2019 to around 35 percent in the last year of our simulation period. Our model predicted a rather pessimistic development of trend total factor productivity. According to the simulation, trend TFP would decline by 0.1 to 0.2 percent p.a. As we regarded this as too pessimistic, we exogenously raised trend TFP via an add factor such that it increases by around 2.3 percent per year on average during the simulation period.

## Policy simulations

In this section we analyse the effectiveness of fiscal policies in Slovenia. For this purpose, we are interested in the deviations of important macroeconomic aggregates from the baseline simulation described in the previous section. We perform eight fiscal policy simulations. As in the baseline, the simulation period runs from 2020 to 2030, with the policy measures implemented from 2020 onwards.

We consider four spending instruments and three tax rates. In addition, we analyse the effects of an increase in the proportion of people with tertiary education in the labour force. Due to a lack of adequate time series data, our model does not contain a specific instrument which relates directly to the education level of the population, such as the number of teachers at secondary schools or the amount of public spending on universities. We assume that the share of people with tertiary education can be influenced by spending measures rather than changes to tax rates. Hence, we count this scenario as a spending measure.

For the simulations we consider the following instruments:

(i) GN:Government consumption, nominal.
(ii) TRANSFERS:Transfers, nominal.
(iii) GINVN:Public investment, nominal.
(iv) GERD:Government expenditure on R&D, nominal.
(v) LFTER:Proportion of people with tertiary education in the labour force.
(vi) VAT:Value added tax rate.
(vii) INCTAX:Personal income tax rate.
(viii) SOCEMP:Employees' social security contribution rate.

For each instrument, we run one separate simulation, i.e. in each simulation only one instrument is altered while for all other instruments the baseline settings are taken.

For the first scenario we assume that from 2020 to 2030 public consumption (GN) is 100 million euros p.a. (25 million euros per quarter) higher than in the baseline. In the second simulation this change is applied to transfers to private households (TRANSFERSN). In the third and fourth simulation, respectively, GINVN and GERD are raised by 25 million euro per quarter or 100 million euros per year over their baseline values. In the fifth scenario, the proportion of people with tertiary education is increased by 0.5 percentage points with respect to the baseline. In the simulations focussing on the revenue side, the value added tax rate is reduced by 1 percentage point from 2020 onwards while in the remaining two simulations the income tax rate and the employees’ social security contribution rate, respectively, are reduced by 0.5 percentage points relative to the baseline.

By definition, public consumption and transfers initially trigger pure demand effects, either directly or via private consumption. Public investment does not only have the demand effect by being part of the GDP expenditure identity (as public consumption) but in addition it raises potential output via the capital stock. Furthermore, the investment ratio influences TFP; thus public investment has two impacts on potential GDP. Public R&D spending also influences total factor productivity and is also part of investment; hence this spending category initiates both demand and supply effects as well. The difference between the impacts of GINV and GERD is that the former affects the TFP only indirectly via the investment ratio while the latter also has a direct effect on total factor productivity.

Ceteris paribus, a higher VAT rate reduces disposable income and hence negatively influences private consumption. Changes in the income tax rate influence the tax wedge, i.e. the difference between gross and net wages. A higher tax wedge has negative effects on both labour demand and labour supply, which is another supply-side policy effect. Increases in the income tax rate, in addition, reduce disposable income. Finally, the social security contribution rate influences the tax wedge and disposable income in the same way as the income tax rate. In addition, changes in employees’ social security contributions also influence employers’ contributions as employers’ and employees’ social security contributions are linked via a behavioural equation.

The following figures show the resulting absolute deviations from the baseline of important macroeconomic aggregates which are generally regarded as policy targets (real GDP level and growth, potential GDP, CPI level, employment, unemployment rate, debt-to-GDP ratio) in the various policy simulations. In order to keep the figures legible, the scenarios targeting the expenditure and revenue sides of the budget are shown in separate figures. The names of the scenarios as indicated in the legends correspond to the policy instruments as mentioned above.

**Fig. 1:**
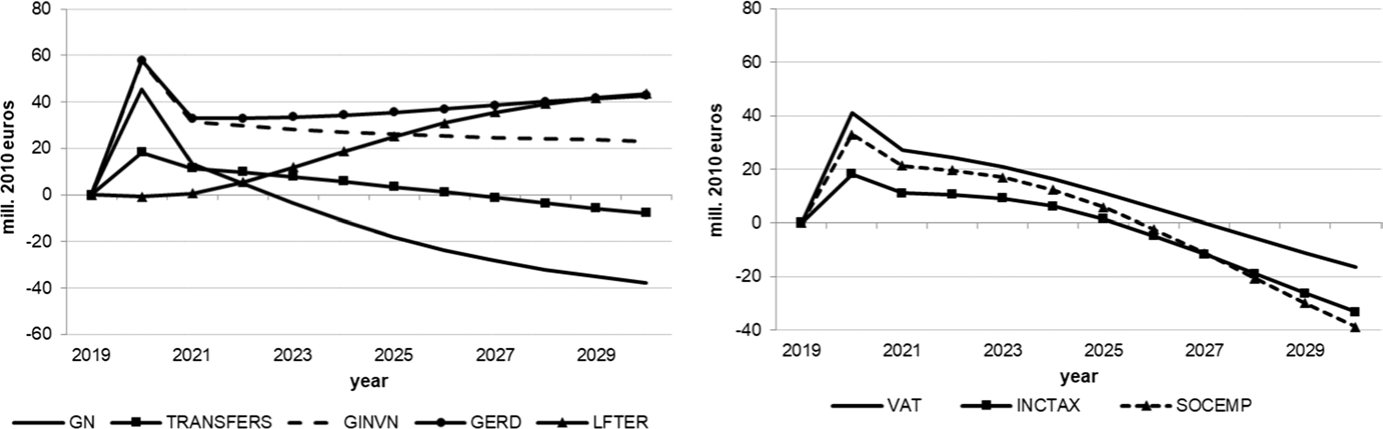
Real GDP. *Source* authors’ calculations and illustration

**Fig. 2:**
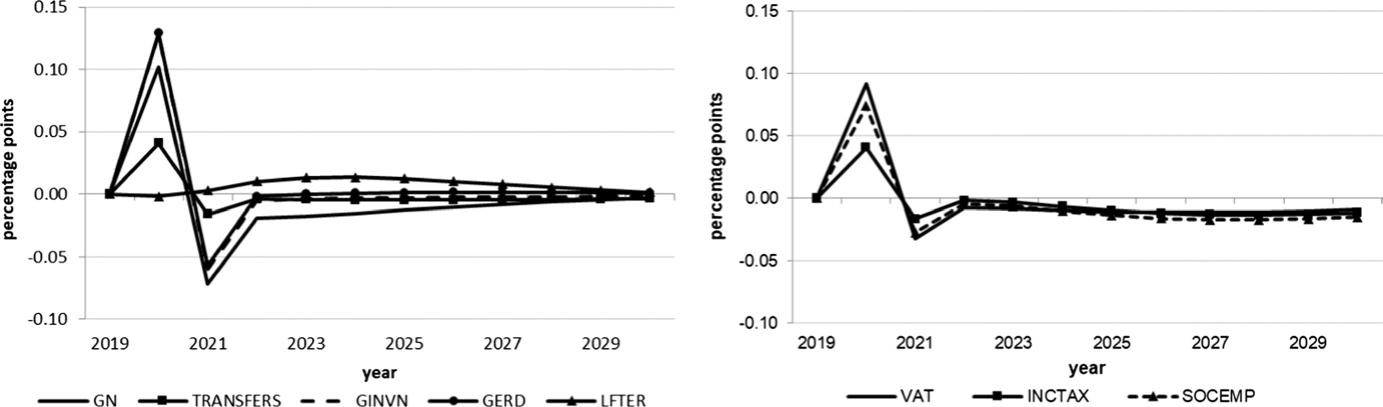
Real GDP growth. *Source* authors’ calculations and illustration

**Fig. 3:**
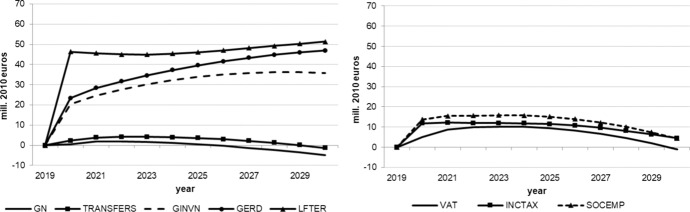
Potential GDP. *Source* authors’ calculations and illustration

As we assumed the change in each of the policy instruments (increases in spending, decreases in taxes) to be approximately equal in size in terms of 2020 euros, we can compare the effectiveness of each of them over time. Figure 1 shows that three instruments from the expenditure side lead to permanently higher real GDP: government spending on R&D (GERD), measures to improve human capital (LEFTER), and government investment (GINV). Conversely, government consumption (GN), transfers (TRANSFERS) and the three tax measures initiate smaller and, more importantly, only relatively short-lived increases in output, with crowding-out effects for public consumption after three years, transfer payments after seven years, income taxes (INCTAX) and social security contributions (SOCEMP) after six years. The instruments with long-run effects are those that address the supply side and increase total factor productivity and, hence, potential output, in addition to aggregate demand. This is also visible in Fig. 3. These effects are strongest for expenditure relating to R&D and tertiary education, in agreement with growth theory, which predicts permanent growth effects primarily from technical progress, to which these two instruments are strongly related. Public investment increases the capital stock, and therefore also potential output, but these increases fall over time due to the diminishing marginal productivity of capital. None of the fiscal policy instruments under consideration exerts a permanent effect on the growth rate of real GDP (Fig. 2), as expected from neoclassical growth theory.

**Fig. 4:**
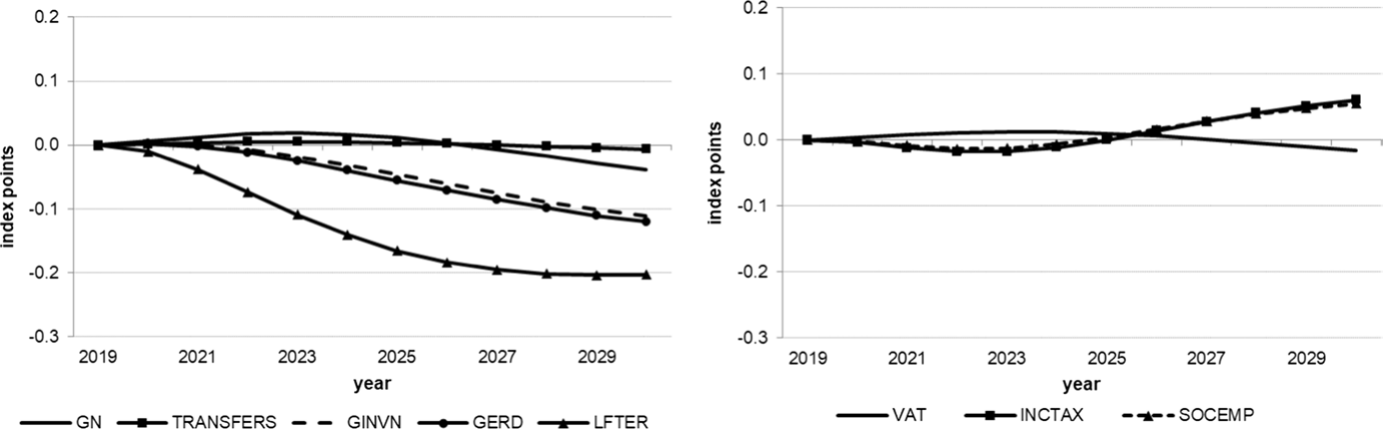
CPI level. *Source* authors’ calculations and illustration

Figure 4 shows that the effects on prices are very small, particularly for the tax measures and increases in transfers. The other instruments, although applied in an expansionary way, lead to a slightly lower price level and (temporarily) lower inflation. This, at first glance, somewhat unexpected result can be explained by the relative size of the supply-side versus demand-side effects: potential output increases more than actual GDP, which implies that the supply-side effect dominates the demand-side effect. For the investment variables (GINV, GERD and LEFTER), this effect is more pronounced due to their impact on the capital stock. However, it also holds for the instruments affecting public or private consumption since the elasticity of imports with respect to GDP is well above one according to the estimated import equation, which dampens the GDP effect (but not the potential output effect) of expansionary fiscal policies.

**Fig. 5:**
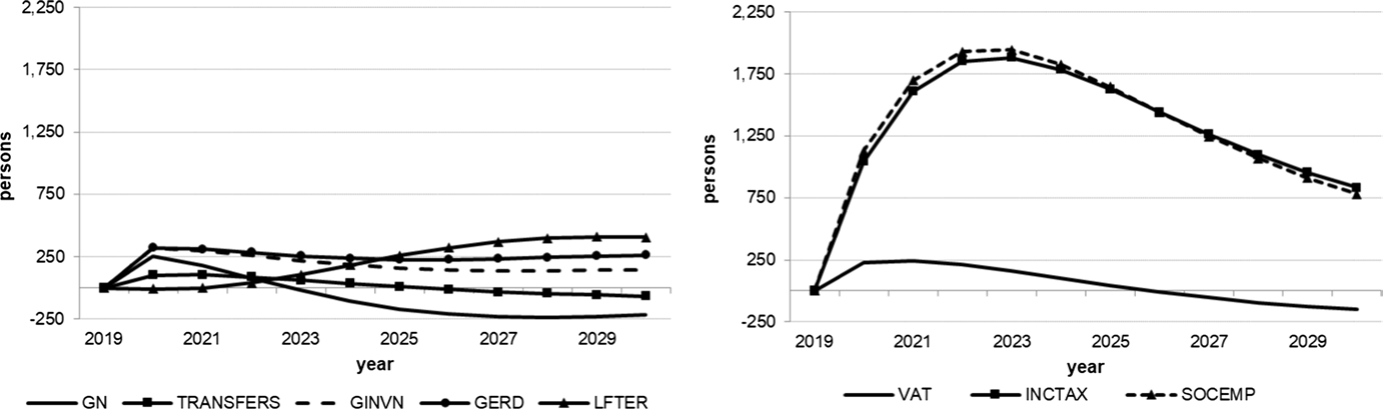
Employment. *Source* authors’ calculations and illustration

**Fig. 6:**
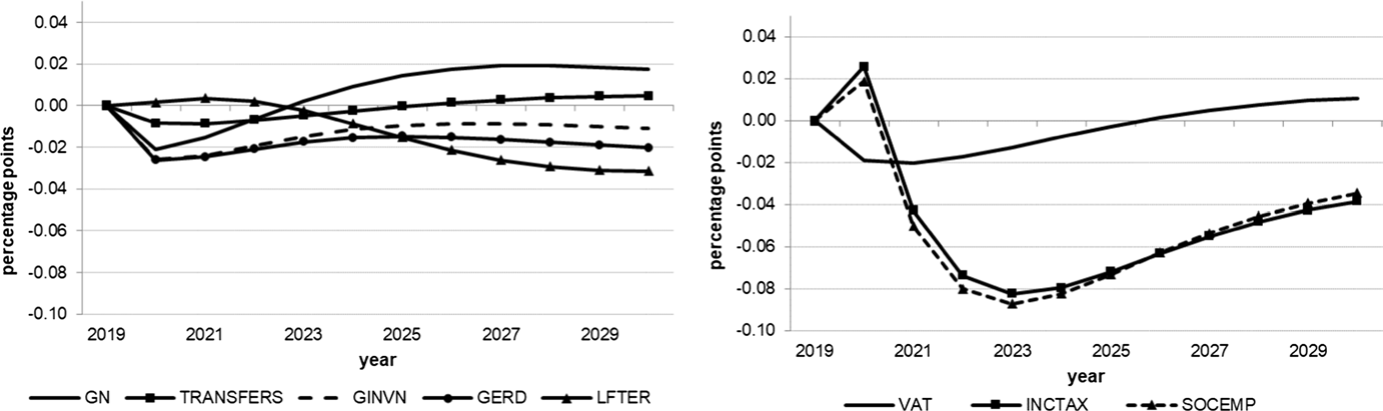
Unemployment rate. *Source* authors’ calculations and illustration

While the tax policy measures considered here have only small effects on the goods market, they impact the labour market more strongly. As Figs. 5 and 6 show, the labour market is affected by the tax measures in particular. On the expenditure side, transfers have only very minor and transitory effects on employment while public consumption effects even turn negative after three years. Again, supply-side measures exert stronger, longer lasting effects which increase over time, especially those measures enhancing R&D and tertiary education. Nevertheless, all of these effects are relatively small in terms of additional employment and reduced unemployment as compared to direct tax reductions. These instruments generate more than six times as many additional jobs as even the most effective expenditure measure, although this effect decreases after four years. From this it can be concluded that policy makers will have to reduce the tax wedge of income tax and social security contributions (payroll related costs) in order to increase employment and decrease unemployment. The small transitory increase in the unemployment rate in the first year (Fig. 6) is due to the fact that labour supply reacts more quickly to the reduction in tax rates than labour demand.

**Fig. 7:**
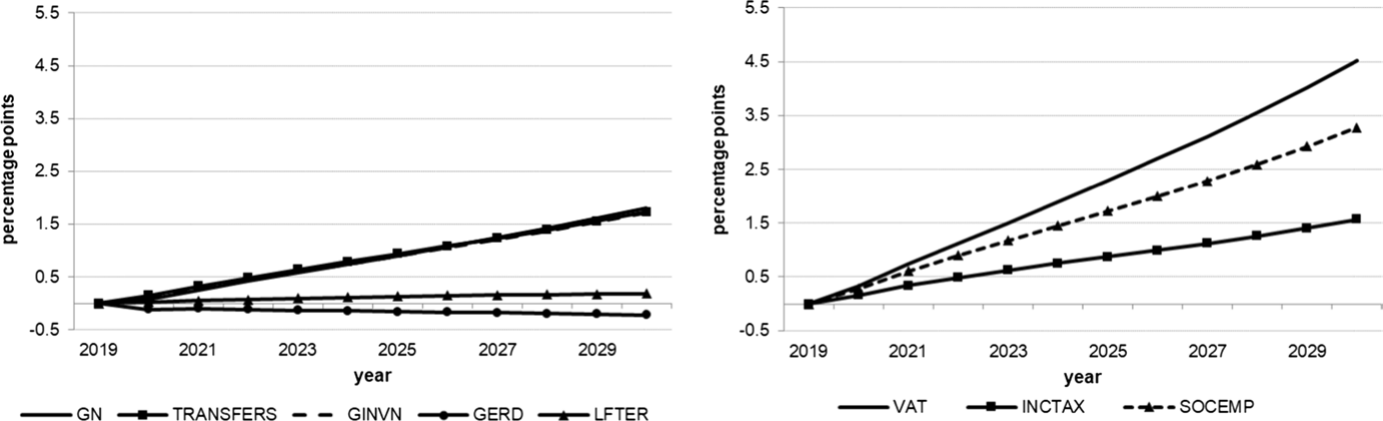
Debt-to-GDP ratio. *Source* authors’ calculations and illustration

Finally, Fig. 7 shows the effects on public debt in relation to nominal GDP. Recall that the immediate effect of each measure on the public budget and, hence, the first round effect (in 2020) on the budget balance is assumed to be approximately the same for each measure. Over time, however, the costs develop differently in terms of a higher debt-to-GDP ratio. When it comes to the sustainability of public finances, the clear winner is expenditure related to R&D, with human capital stimulation coming second. The loser is the reduction in VAT. Given its low effectiveness with respect to output and especially employment, this instrument seems to be rather unattractive. Instead, if containing public debt within the limits prescribed by the EU Stability and Growth Pact is required, an increase in the VAT rate to finance income tax reductions and additional supply-side related expenditure may be a reasonable policy mix.

## Covid-19 effects

Given the fact that the Covid-19 pandemic is affecting Slovenia like nearly all countries in the world, the reader may question the analysis presented in Sects. 3 and 4 by asking why determine policy multipliers for a scenario which did not occur and will not occur in the near future. The reason is twofold: First, we consider the policy effects in “normal times”, which may include recessions such as the Great Recession but not a catastrophic event such as Covid-19. Second, at the time of finishing this study (beginning of December 2020), we are in the midst of the Covid-19 crisis: only very tentative results can be obtained as the length and degree of the crisis and its economic impacts cannot be even approximately assessed. Moreover, it is not yet clear what fiscal policies in Slovenia and—even more uncertain—in the Euro Area will look like and how they will impact on a national economy like the Slovenian. Nevertheless, we attempt to sketch a possible scenario of possible macroeconomic effects of the pandemic. To do so, we start from the baseline solution of the model and consider some exogenous shocks which we believe are to be expected or are already in force in Slovenia.

In Slovenia, the first victim of the virus (the first person to be tested positively) was identified on March 4, 2020, with increases in the cumulative number of infected persons to low 4-digit numbers one month later, where they stayed with only small increases until the start of October. The first case of death from Covid-19 occurred on March 14. Then, after the end of the summer vacations and associated travel activities, the number of daily infections increased to 4-digit numbers, resulting in a cumulative number of infections of over 80,000 at the beginning of December 2020. Thus, like in many other European countries, a first wave occurring in spring was followed by a second, and much stronger, wave in fall.

The Slovenian government reacted to this development as follows: on March 13, the epidemic was declared and the Slovenian Parliament adopted several emergency laws (approved by the European Commission, where required), supported by measures involving the Slovenian Export and Development Bank and the Slovene Enterprise Fund. Starting on March 16, all educational institutions were closed, all public transport and all “unnecessary” services in the country were suspended, and all hotels, restaurants, and bars were closed. On March 20 de facto quarantine (with some exceptions) was established (Damijan 2020). Financial support to enterprises included direct corporate financing in the form of long-term loans with favourable interest rates, direct and indirect funding of small and medium enterprises to cover their costs during the epidemic, the financing of investments and working capital for the sustainable growth of tourism, funds for health care institutions and establishments, microloans for companies in areas with high unemployment and in border areas, and guarantees for bank loans with an interest rate subsidy.

The reaction of the Slovenian government to the first wave in March proved to be successful, as the numbers of new infections could be kept low, not only during the lockdown but also over the entire summer. In fact, during this period, Slovenia was among the best European countries in terms of infections per 100,000 inhabitants. At that time, economic forecasts (Damijan 2020; Bank of Slovenia 2020, and IMAD 2020) were rather optimistic, with a projected decline in real GDP by 6–7 percent in 2020 and a recovery of about the same size in the following year.

Unfortunately, the situation changed dramatically with the advent of the second wave of the pandemic. Although nearly all European countries were affected by that wave, Slovenia was hit particularly hard, with the numbers of deaths per 100,000 inhabitants being among the 10 highest worldwide in December. The Slovenian government reacted in a similar way to the first wave: on October 18, a state of emergency was declared for 30 days (which was subsequently tightened and prolonged until mid-January 2021), involving a nationwide curfew from 9 pm to 6 am (with few exceptions), severe restrictions on travel, the suspension of public transport, distance learning in all educational institutions, an obligation to wear masks everywhere outside the home, and a ban on gatherings and events; most non-essential businesses were closed. Nevertheless, the numbers of newly infected persons per day in Slovenia were still in the 4-digit range at the beginning of December 2020.

Although the epidemic is still expanding in Slovenia and no current economic forecast has taken this into account, we dare provide a first estimate of the macroeconomic effects of this process. The last available forecast for world trade volume is from October 6 from the WTO, estimating –9.2% for 2020 and + 7.2% for 2021, with the latter probably being too optimistic, even if vaccination has a positive effect on the situation in 2021. The same is true for the autumn forecast (IMAD 2020a and 2020b), predicting GDP growth in Slovenia of –6.7 for 2020 and + 5.1 for 2021.

**Fig. 8:**
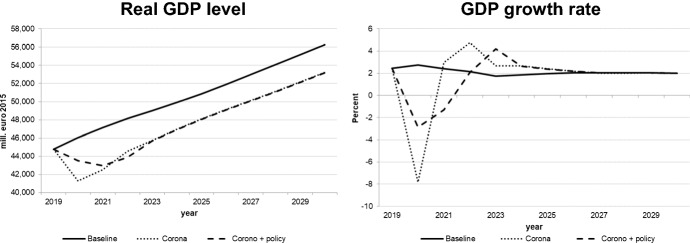
Real GDP (level and growth rate). *Source* authors’ calculations and illustration

**Fig. 9:**
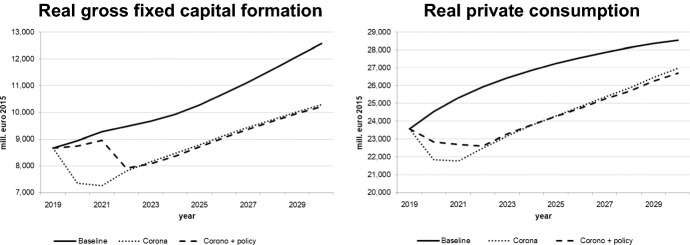
Consumption and investment. *Source* authors’ calculations and illustration

**Fig. 10:**
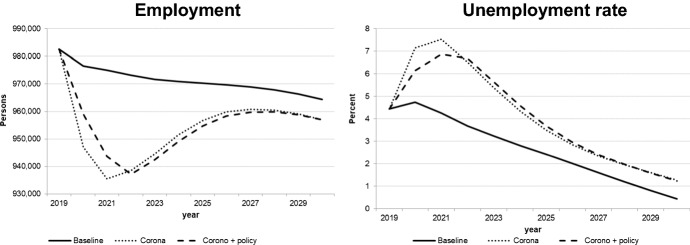
Labour market. *Source* authors’ calculations and illustration

**Fig. 11:**
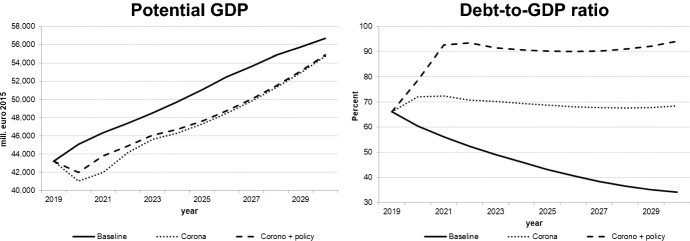
Potential output and debt-to-GDP ratio

We decided to interpret the Covid-19 shock as a combination of demand and supply shocks. The extent of the negative exogenous shocks on real private consumption, private investment, and exports as well as on potential output was calibrated (in the quarterly version of the model) so as to match the realized values of these variables for the first wave (2020q2) as closely as possible; for the second wave (2020q4 and 2021), we assumed slightly stronger impacts which would diminish gradually in 2021. This implies a more pessimistic estimate of the Slovenian economy over the first few months of 2021, even if vaccination is possible in 2021. For the following years, growth from the lower level of 2021 with similar rates as in the baseline solution was assumed. The figures for the key macro variables illustrated in Figs. 8, 9, 10, 11 show the results of this simulation denoted by “Corona”. A second simulation takes government policy measures into account as announced so far; we assume that government expenditures to continue in 2021 to mitigate decreases in output and employment. This simulation is labelled “Corona + policy”. As expected, simulated real GDP falls in 2020 and remains below the baseline until the end of the simulation period, with similar effects on potential GDP. The effects on private consumption and investment are qualitatively similar, and it takes until the middle of the next decade to regain pre-crisis levels. Although GDP growth resumes baseline values by then, the levels of GDP and its components remain below baseline until the end of the simulation period; hence the pandemic implies a permanent output loss. For employment and unemployment, on the other hand, convergence toward the baseline occurs in the late 2020s. The main difference achieved by the fiscal policy measures is a delay in the decline in investment and employment and a smoother path for GDP, at the expense of a permanent (in the absence of consolidation measures) increase in the public debt-to-GDP ratio by about 20 percentage points.

Of course, it must be stressed that these scenarios have been constructed under a very high degree of uncertainty both with respect to future epidemiological developments and international and national policy reactions, especially in the EU and the Euro Area in particular. If we consider the results of the calculation of the multipliers, in any case it would be advisable for the Slovenian government to supplement its fiscal policy measures with some measures increasing expenditures on research and development and on education, as these have the strongest effects on aggregate demand and supply.

## Conclusions

In this paper, we investigated the effectiveness of fiscal policy measures to increase output and reduce unemployment in the case of a fall in aggregate demand. Despite Slovenia’s very favourable economic development in the past two years, this question boils down to which fiscal policy measures would be adequate should an event similar to the Great Recession occur again. We used the SLOPOL10 econometric model of the Slovenian economy to simulate the effects of different expansionary fiscal policy measures on the revenue and expenditure sides.

Our results show that public spending measures which entail both demand- and supply-side effects, i.e. public investment and especially spending on R&D and tertiary education, are more effective at stimulating real GDP than pure demand-side measures. Measures that improve the education level of the labour force are very effective at stimulating potential GDP. Employment can be most effectively stimulated by cutting the income tax rate and the social security contribution rate. Higher spending on research and development only has negligible effects on the debt-to-GDP ratio while all the other fiscal policy measures that we considered lead to higher public debt. Due to the high elasticity of imports with respect to demand, pure demand-side effects on real variables are small, showing that a small open economy like Slovenia only has very little scope for influencing macroeconomic developments with pure demand management policies. We also provide tentative simulations (without and with active fiscal policy) for the current Covid-19 pandemic and its economic effects.

Of course, it would be premature to suggest strong and precise recommendations for the policy makers in Slovenia based on just one model specification. It should be noted, however, that in a recent study with a New Keynesian DSGE model, Sims and Wolff (2018) show that countercyclical fiscal policies are more successful when using productive public investment instead of public consumption. Our results also clearly support the theory and empirical evidence that supply-side policies strengthening potential GDP bring about the best results in terms of stimulating economic growth and employment without putting too much additional strain on public finances. Moreover, the labour market can best be influenced positively by reducing the tax wedge.
